# Cocaine use in university students: relationships with demographics, mental health, risky sexual practices, and trait impulsivity

**DOI:** 10.1017/S1092852920001492

**Published:** 2021-10

**Authors:** Samuel R. Chamberlain, Katherine Lust, Jon E. Grant

**Affiliations:** 1 Department of Psychiatry, University of Cambridge, Cambridge, United Kingdom; 2 Cambridgeshire and Peterborough NHS Foundation Trust (CPFT), Cambridge, United Kingdom; 3 Boynton Health Service, University of Minnesota, Minneapolis, Minnesota USA; 4 Department of Psychiatry & Behavioral Neuroscience, University of Chicago, Chicago, Illinois, USA

**Keywords:** stimulant, illicit, drugs, addiction, impulsivity, compulsivity

## Abstract

**Background:**

Cocaine is increasingly used on a recreational basis by the general population with potential implications for mental health. The aim of this study was to assess how common cocaine use is, and its mental health associations, in a large sample of university students.

**Methods:**

Approximately 10 000 university students were invited to take part in an online survey, which assessed the use of cocaine (ever or past year), alcohol and drug use, mental health issues, and impulsive and compulsive tendencies. Group differences in demographic and clinical characteristics were characterized.

**Results:**

A total of 3520 university students (57.7% female) completed the survey. Of these, 110 students (3.1%) reported using cocaine in the preceding year, and a further 163 students (4.6%) reported historical use more than a year ago. Cocaine use was associated with more years as a student, lower grade point averages, more use of other drugs, riskier sexual practices, post-traumatic stress disorder, attention deficit hyperactivity disorder, treatment for psychological/emotional problems (including taking prescribed medication), and trait impulsivity. Of these associations, the link with trait impulsivity had the largest effect size.

**Conclusion:**

History of cocaine use appears relatively common in university students; and has a number of untoward associations in terms of mental health, use of other substances, and risky sexual practices. The most marked finding (in terms of effect size) was the link between cocaine use and trait impulsivity, supporting the importance of this construct in seeking out candidate vulnerability markers for use of cocaine and other drugs. Future work should use longitudinal designs to further characterize the nature of these associations.

## Introduction

Cocaine is a stimulant drug with high addictive potential due to its effects on brain dopaminergic reward pathways. It is typically snorted, rubbed into the gums, inhaled as vapor, or dissolved and injected intravenously. After peaking in the 1980s in the United States, recreational use of cocaine declined, but more recently has started to escalate again.^1^ The global prevalence of cocaine use has been estimated at 0.4%, and use of such stimulants is associated with increased risk of death, blood-borne infections, worse mental health (including mood disorders, psychosis, aggression, and suicidality), and untoward physical consequences (such as strokes and heart attacks).^2^ In a multi-criteria decision analysis comparing relative harms of different drugs, crack cocaine (ie, a highly concentrated form of cocaine) ranked highest of all drugs considered in terms of harms to users—higher than heroin. Cocaine (ie, noncrack cocaine) ranked fifth highest in terms of relative harms of different drugs.^3^ In a large survey of people who use recreational drugs, across all the drugs considered, cocaine ranked second highest in terms of average perceived harm, with only opiates (or prescription analgesics more widely) ranking higher.^4^

Adolescence and young adulthood constitute a crucial neurodevelopmental period when the brain may be particularly susceptible to untoward effects of illicit drugs such as cocaine.^5^ ^,^^6^ Furthermore, in young adulthood, people are typically undertaking crucial periods of education or establishing themselves in careers, and forming long-term relationships. As such, untoward effects of illicit drugs in young people can have profound long-term functional consequences. In 2015, a U.S. survey found that the majority of people using cocaine for the first time were young adults.^1^

As noted, cocaine use has been associated with a number of mental health difficulties in general, and several studies have examined young adults. In a sample of 421 university students in the United States, 6.4% reported past-year cocaine use.^7^ Cocaine use was significantly correlated with functional impairment across multiple domains (eg, social/personality/reputation and friendships), weight loss, being unhappy due to drug use, and being more impulsive when using drugs (eg, doing regretted impulsive acts, making harsh/cruel comments to others, or taking foolish risks).^7^ In a large study using data from the National Education Longitudinal Study (United States) (n = 4008), using regression models, lifetime cocaine use was significantly associated with fewer years of education being completed.^8^ In longitudinal data from the College Life Survey (United States) (n = 1253), by year 4, 36% of students had been exposed to at least one potential opportunity to take cocaine (lifetime), and 13% had used cocaine at least once.^9^ Cocaine use was statistically associated with being male, white, and with other drug use (including alcohol use disorder). Cocaine use was not associated with socioeconomic status.

In view of the escalating use of cocaine reported in young people more recently, coupled with gaps in the literature regarding aspects of mental health associations, the aim of the current study was to examine (1) the extent of past-year and historical use of cocaine in a large sample of university students and (2) demographic and clinical associations with cocaine use in this sample. We included questionnaire-based measures of impulsivity and compulsivity, since these concepts have been implicated in different stages of addiction.^10^ Data were also collected about risky sexual practices, this being an important public health issue, since drug use may lead to this. We hypothesized that cocaine use would be associated with higher levels of depression, anxiety, other substance use; with elevated trait levels of impulsivity; and with riskier sexual practices.

## Methods

### Survey design

Researchers at the Department of Psychiatry and Behavioral Neuroscience at the University of Chicago and Boynton Health Services at the University of Minnesota jointly developed the survey, which was completed over the Internet by participants. All study procedures were conducted in accordance with the Declaration of Helsinki and the University of Minnesota’s Institutional Review Board approved the study.

### Participants

Approximately 10 000 students at a large Midwestern university were selected and invited to take part in the study. These students were selected using a computer-generated random selection of all students at the institution.

Participants first read the information sheets and provided consent using an online interface—they were assured that participation was confidential. Students were informed that those completing the survey would be entered into a prize draw whereby 10 students would be randomly chosen to receive prizes: 3 would win tablet computers, 4 would win $250 gift certificates, 2 would win $500 gift certificates, and 1 would win a $1000 gift certificate. Participants were assured that their contact details for the prize draw would be stored completely separately from their survey responses, in order to ensure their responses were kept completely confidential. Following provision of consent, the survey was presented.

### Assessments

The survey comprised 156 questions and took approximately 30 minutes to complete. Cocaine use (any type) was measured by asking participants if they had (1) never used cocaine, (2) had used cocaine in the past but not in the past year, or (3) had used cocaine in the past year. Response to this question was used to classify participants into the three study groups, hereafter referred to as controls (1), past year cocaine users (2), and historical cocaine users (3), respectively. The survey collected relevant demographic information, clinical information, and sexual health information.

### Drug and alcohol use

Participants were asked if they had ever used an illicit drug (binary); and were asked about whether they had used the following in the past 12 months (each a binary response): amphetamines, heroin, hallucinogens, marijuana or hashish, prescription opioid pain medication, or sedatives. In addition to use of drugs and alcohol, participants completed the *Alcohol Use Disorders Identification Test (AUDIT)* (score of ≥8 indicating potentially harmful alcohol use)^11^; and the *Drug Abuse Screening Test (DAST-10)* (score of 3 indicating a positive screen for a drug use disorder).^12^ ^,^^13^

### Mental health problems

Participants completed the following previously validated questionnaires: *Patient Health Questionnaire (PHQ-9)* (score of ≥10 indicating depressive symptoms of moderate or higher severity)^14^; *Generalized Anxiety Disorder 7 (GAD-7)* (score of 10 or greater indicating clinically significant anxiety)^15^; *Primary Care PTSD Screen (PC-PTSD)* (score of ≥3 indicating probable post-traumatic stress disorder, PTSD)^16^; *Adult ADHD Self-Report Scale (ASRS-v1.1) Part A* (6 questions screening for attention-deficit/hyperactivity disorder [ADHD])^17^ ^,^^18^; *Minnesota Impulsive Disorders Interview (MIDI)* (binge eating disorder and gambling disorder)^19^ ^,^^20^; and the *Rosenberg Self-Esteem Scale (RSES)* (score <15 indicating low self-esteem).^21^

### Impulsivity and compulsivity

Impulsivity was assessed using the *Barratt Impulsiveness Scale, Version 11 (BIS-11)* (yielding three dimensions of impulsivity—attentional, motor, and nonplanning)^22^ ^,^^23^; and compulsivity was assessed using the *Cambridge-Chicago Compulsivity Trait Scale (CHI-T)* (yielding a total compulsivity score).^24^

### Data analysis

Effects of group (past year cocaine users, historical cocaine users, and controls) were explored using likelihood ratio tests for categorical variables, and analysis of variance for continuous variables. Effect sizes were also reported using Cramer’s V or Cohen’s D respectively. SPSS was used for all statistical analyses (version 24; IBM Corp). Statistical significance was defined as *P* < .05, Bonferroni corrected for the number of measures in each table (ie, at the level of type of measurement).

Missing data were missing completely at random (MCAR) and the analysis was conducted using list-wise deletion. Because this was a large sample, where power was not an issue, the assumption of MCAR was satisfied and list-wise deletion was thus appropriate.

## Results

A total of 3520 university students (57.7% female) completed the survey. Of these, 110 students (3.1% of the total sample) reported past year cocaine use, and a further 163 students (4.6% of the total sample) reported historical use (ie, use of cocaine more than 1 year ago).

The demographic features of the groups are shown in Table 1. It can be seen that cocaine use was more common in older undergraduate students, and was associated with lower grade point averages. Racial-ethnic group (likelihood of being Caucasian) did not differ significantly between groups. Cocaine use was not significantly related to gender. In terms of marital status, current cocaine use was associated with a lower likelihood of being married, whereas past cocaine users did not differ markedly from controls in terms of likelihood of being married.

**Table 1.** Demographics of University Students Based on Cocaine Use Status

Variable	Students Who Currently Use Cocaine (n = 110)	Students Who have Used Cocaine in the Past (n = 163)	Students Who have Never Used Cocaine (n = 3247)	Likelihood Ratio *χ* ^2^	*P* Value	Effect Size Cramer’s V
Gender						
Male	36.4 (40)	44.2 (72)	35.3 (1144)	LR = 11.34	.087	.041
Female	57.3 (63)	49.7 (81)	58.2 (1886)	df = 6		
Trans/GenderQueer	3.6 (4)	2.5 (4)	1.4 (44)			
No answer	2.7 (3)	3.7 (6)	5.2 (167)			
Student status						
First year undergrad	10.9 (12)	2.5 (4)	17.2 (558)	LR = 90.394	<.001*	.115
Second year undergrad	10.9 (12)	8.6 (14)	15.4 (499)	df = 14		
Third year undergrad	15.5 (17)	17.8 (29)	15.6 (505)			
Fourth year undergrad	32.7 (36)	16.6 (27)	13.8 (447)			
Fifth year or more undergrad	10.9 (12)	9.2 (15)	3.8 (124)			
Graduate—Master’s	10.9 (12)	20.9 (34)	14.7 (477)			
Graduate—Doctoral/Prof	8.2 (9)	23.9 (39)	18.9 (612)			
Non-degree seeking	0.0 (0)	0.6 (1)	0.6 (19)			
Race/ethnicity, Caucasian	85.0 (91)	79.4 (123)	75.0 (2307)	LR = 7.535	.023	.045
				df = 2		
Relationship status						
Single	44.5 (49)	30.1 (49)	45.8 (1483)	LR = 25.125	<.001*	.06
Dating	45.5 (50)	50.3 (82)	38.2 (1239)	df = 6		
Engaged/married	7.3 (8)	17.8 (29)	15.1 (489)			
Other	2.7 (3)	1.8 (3)	0.9 (29)			
College GPA						
Below 1.50	1.8 (2)	0.0 (0)	0.2 (5)	LR = 52.618	<.001*	.095
1.50-2.49	3.6 (4)	3.1 (5)	1.5 (47)	df = 8		
2.50-2.99	18.2 (20)	12.9 (21)	8.0 (256)			
3.00-3.49	47.3 (52)	38.0 (62)	32.7 (1047)			
3.50-4.00	29.1 (32)	46.0 (75)	57.7 (1845)			

All numbers are % (N) unless otherwise stated.

* *P* value significant with Holm-Bonferroni sequential correction (critical *P* = .05/5 = .01).

Table 2 shows drug and alcohol use status in the study groups. Cocaine use was associated with more nicotine use (including e-cigarette use), higher endorsement of using other illicit drugs over the lifetime (this was significant for all categories of illicit drugs that were measured), higher AUDIT and DAST scores indicative of substance use disorders, higher use of drugs to lose weight, and higher likelihood of previous treatment for drug/alcohol problems.

**Table 2.** Drug and Alcohol Use of University Students Based on Cocaine Use Status

Variable	Students Who Currently Use Cocaine (n = 110)	Students Who have Used Cocaine in the Past	Students Who have Never used Cocaine (n = 3247)	Likelihood Ratio χ^2^^a^	*P* Value	Effect Size Cramer’s V
Nicotine use	86.4 (95)	89.0 (145)	35.7 (1158)	LR = 297.42	<.001*	.285
E-cigarette use	58.7 (64)	55.6 (89)	14.4 (468)	LR = 241.72	<.001*	.302
Illicit drug use (lifetime)	100 (110)	100 (163)	34.0 (1099)	LR = 549.41	<.001*	.363
Drug use (past 12 mo)						
Amphetamines-non prescription	13.6 (15)	2.5 (4)	0.4 (14)	LR = 68.24	<.001*	.241
Amphetamines –prescription (not mine)	60.9 (67)	24.5 (39)	3.7 (120)	LR = 325.88	<.001*	.436
Heroin	6.4 (7)	1.3 (2)	0.1 (4)	LR = 36.31	<.001*	.182
Hallucinogens	51.8 (57)	14.2 (23)	2.7 (86)	LR = 258.47	<.001*	.415
Marijuana or hashish	84.3 (96)	64.2 (104)	24.0 (776)	LR = 288.32	<.001*	.304
Rx opioid pain medication	25.5 (28)	10.1 (16)	1.0 (31)	LR = 146.55	<.001*	.318
Sedatives	19.1 (21)	10.0 (16)	1.1 (36)	LR = 103.00	<.001*	.251
AUDIT score ≥8 (%)	83.6 (92)	52.5 (85)	21.2 (687)	LR = 248.27	<.001*	.29
DAST-10 score ≥3 (%)	46.4 (51)	44.2 (72)	5.1 (165)	LR = 312.60	<.001*	.39
Has been treated for drug/alcohol use problems						
Yes	7.4 (8)	15.7 (25)	0.9 (30)	LR = 94.00	<.001*	.243
Has used drugs in order to lose weight						
Yes	30.9 (21)	24.7 (19)	6.4 (94)	LR = 54.80	<.001*	.222

All numbers are % (N) unless otherwise stated.

Abbreviations: AUDIT, Alcohol Use Disorders Identification Test; DAST, Drug Abuse Screening Test.

a Degree of freedom = 2.

* *P* value significant with Holm-Bonferroni sequential correction (critical *P* = .05/14 = .004).

In terms of sexual health characteristics in the groups, these are shown in Table 3. Cocaine use was associated with higher likelihood of being sexually active, earlier age of first sexual experience, and less frequent use of barrier protection. Cocaine use was not significantly related to compulsive sexual behavior (though it should be noted that occurrence of compulsive sexual behavior in the sample was relatively infrequent in the whole sample).

**Table 3.** Sexual Health in University Students Based on Cocaine Use Status

Variable	Students Who Currently Use Cocaine (n = 110)	Students Who have Used Cocaine in the Past	Students Who have Never used Cocaine (n = 3247)	Statistic Likelihood Ratio	*P* Value	Effect Size Cramer’s V
Has been sexually active						
Yes	94.5 (104)	96.3 (156)	71.1 (2281)	LR = 103.22	<.001*	.148
				df = 2		
Age at first sexual activity with another						
<11 y	1.9 (2)	1.3 (2)	0.7 (17)	LR = 110.74	<.001*	0.152
12--14 y	14.4 (15)	18.6 (29)	4.8 (110)	df = 8		
15--17 y	59.6 (62)	53.8 (84)	40.0 (911)			
18--20 y	23.1 (24)	21.2 (33)	40.8 (928)			
21 y or older	1.0 (1)	5.1 (8)	13.6 (310)			
Frequency of physical barrier use						
<50% of the time	49.0 (51)	54.5 (85)	37.3 (847)	LR = 48.22	<.001*	.094
50%--75% of the time	11.5 (12)	13.5 (21)	8.8 (199)	df = 6		
76%--95% of the time	21.2 (22)	14.1 (22)	16.4 (373)			
96%--100% of the time	18.3 (19)	17.9 (28)	37.6 (854)			
Compulsive sexual behavior?						
Positive screen	6.1 (6)	7.6 (12)	3.5 (106)	LR = 7.16	.028	.052
				df = 2		

All numbers are % (N) unless otherwise stated.

* *P* value significant with Holm-Bonferroni sequential correction (critical *P* = .05/5 = .0125).

For impulsive behavior and mental health histories (Table 4), cocaine use was associated with higher likelihood of PTSD, higher likelihood of ADHD, more previous treatment for psychological/emotional problems, and higher likelihood of taking prescribed psychiatric medication. Cocaine use status was not significantly related to caffeine use, gambling disorder, binge-eating disorder, current depression/anxiety scores, nor to self-esteem levels.

**Table 4.** Impulsive Behaviors and Psychiatric History of University Students Based on Cocaine Use Status

Variable	Students Who Currently Use Cocaine (n = 110)	Students Who have Used Cocaine in the Past (n = 163)	Students Who have Never Used Cocaine (n = 3247)	Statistic Likelihood Ratio	*P* Value	Effect Size Cramer’s V
Amount of caffeinated soft drinks consumed over the past week n (%)						
Never	40.4 (44)	46.3 (74)	48.4 (1542)	LR = 23.12	.01	.069
1--2 drinks	27.5 (30)	31.3 (50)	32.5 (1036)	df = 10		
3--6 drinks	22.0 (24)	11.3 (18)	12.5 (399)			
7--12 drinks	3.7 (4)	5.6 (9)	4.4 (141)			
13--23 drinks	2.8 (3)	4.4 (7)	1.4 (46)			
24 or more drinks	3.7 (4)	1.3 (2)	0.7 (21)			
Gambling disorder?						
Positive screen	1.9 (2)	1.9 (3)	0.3 (9)	LR = 8.75	.013	.067
				df = 2		
Binge eating disorder?						
Positive screen	3.7 (4)	3.2 (5)	2.4 (74)	LR = 1.04	.595	.019
				df = 2		
PHQ-9 score ≥10 (%)	8.6 (9)	4.5 (7)	4.5 (139)	LR = 3.15	.207	.034
				df = 2		
PC-PTSD score ≥3 (%)	26.7 (28)	19.6 (31)	13.7 (430)	LR = 15.07	.001*	.072
				df = 2		
GAD-7 score ≥10 (%)	23.6 (24)	15.3 (24)	17.6 (542)	LR = 2.33	.312	.027
				df = 2		
ADHD						
Positive screen	29.9 (32)	25.0 (39)	16.7 (517)	LR = 16.72	<.001*	.075
				df = 2		

All numbers are % (N) unless otherwise stated.

Abbreviations: ADHD, attention deficit hyperactivity disorder; GAD-7, Generalized Anxiety Disorder 7; PQH-9, Patient Health Questionnaire; PC-PSTD, Primary Care Post-Traumatic Stress Disorder Screen.

* *P* value significant with Holm-Bonferroni sequential correction (critical *P* = .05/10 = .005).

In terms of impulsive and compulsive questionnaire scores (Table 5), cocaine use was associated with higher levels of impulsivity (total scores; and each sub-score), but not with higher levels of compulsivity.

**Table 5.** Impulsivity and Compulsivity of University Students Based on Cocaine Use Status

Variable	Students Who Currently use Cocaine (n = 110)	Students Who have Used Cocaine in the Past (n = 163)	Students Who have Never Used Cocaine (n = 3247)	Statistic ANOVA	*P* Value	Effect Size Cohen’s *d*
Cambridge–Chicago Compulsivity Trait Scale Mean (SD)	9.81 (14.07)	8.55 (13.16)	9.35 (13.54)	*F* (2 3402) = .329	.719	.04
Barratt Impulsiveness Scale (BIS-11) Total Score Mean (SD)	65.98 (9.90)^a^	63.05 (10.45)^b^	59.01 (10.07)^ab^	*F* (2 3175) = 34.12	<.001*	.6714
Attentional impulsiveness mean (SD)	18.09 (3.91)^a^	17.32 (4.37)^b^	16.09 (3.94)^ab^	*F* (2 3268) = 19.55	<.001*	.4922
Non-planning impulsiveness mean (SD)	25.11 (4.39)^a^	24.08 (4.54)^b^	22.80 (4.75)^ab^	*F* (2 3262) = 16.73	<.001*	.4752
Motor impulsiveness mean (SD)	22.97 (4.14)^a^	21.66 (3.79)^a^	20.16 (3.91)^a^	*F* (2 3276) = 35.67	<.001*	.6995

Data refer to mean and (standard deviation [SD]).

ab Post hoc Bonferroni test for significance: The mean difference is significant at the .05 level

* *P* value significant with Holm-Bonferroni sequential correction (critical *P* = .05/5 = .01).

## Discussion

This is perhaps one of the largest studies to date to assess a relatively broad set of demographic and mental health measures in university students, and their association with cocaine use. While cocaine use (and use of other illicit potent stimulants) has been linked to worse mental health in general,^2^ surprisingly little research has focused on young adults. We found that 3.1% of the sample had used cocaine in the past year, and an additional 4.6% had used cocaine historically (ie, total 7.7% of the sample reported cocaine use at some point in life). In the Youth Risk Behavior Surveys (YRBS), lifetime cocaine use was 5.2% of young people in 2015.^25^ The authors found that use appeared to be declining from 1999 to 2009, but then started to increase from 2009 to 2015, drawing attention to the public health importance of the apparent change such that cocaine use was becoming more common. Our data are in keeping with this public health message, since our lifetime rate was relatively high (7.7%) in these young people. Our findings will now be discussed across five distinct domains: demographics, other substance use, sexual health, psychiatric history (including impulsive disorders), and trait impulsivity/compulsivity.

We did not find that cocaine use differed as a function of gender nor ethnic group in our sample (Table 1), militating against the notion that its use is disproportionately more common in Caucasian male university students, and instead highlighting that from a public health perspective it may be important to target screening and interventions irrespective of these demographic variables in young people. More years as a student was associated with higher likelihood of cocaine use, presumably reflecting exposure to drug taking opportunities within university environments, when young people tend to intermix and be exposed to many peer groups. Past year cocaine use was associated with a lower likelihood of being married, whereas historical cocaine use was not. This may indicate that using cocaine may interfere with formation of stable intimate relationships but if one is able to give up using cocaine, then this untoward impact is negated. Last, in terms of demographic characteristics, cocaine use was associated with lower grade point averages, mirroring previous findings in the literature. It should be noted that the significant relationships between cocaine use and demographic characteristics that were observed, were of small effect size.

Cocaine use was associated with elevated rates of the full gamut of use of other illicit drugs, as well as with higher likelihood of using drugs to lose weight, and these results were generally of medium effect size (Table 2). These data may indicate that some individuals have a general propensity toward using drugs irrespective of drug class. We suggest that this propensity likely reflects impulsive tendencies extant prior to drug use (see later discussion also, in terms of trait impulsivity). Impulsivity as a broad concept has been implicated as a vulnerability factor across substance use problems, including in the early stages of pathology (ie, in occasional / recreational drug use)^26^ ^–^^28^ The link with cocaine users having a higher rate of using drugs to lose weight would fit with the stimulant nature of cocaine—stimulants tend to suppress appetite.

For sexual health measures (Table 3), cocaine use was associated with earlier age at first sexual activity, higher likelihood of being sexually active, and lower use of barrier contraception, all with small effect size. We did not observe a significant relationship between cocaine use and compulsive sexual behavior. Reciprocal longitudinal relationships have been found between illicit drug use and risky sexual practices in young people.^29^ These relationships are likely to be complex and differ across individuals. On the one hand, using psychoactive drugs such as cocaine may lead to acute impairment in aspects of cognition (such as impairment in inhibitory control and decision-making) in turn making the individual more likely to engage in risky sexual practices in the moment. On the other hand, engaging in risky sexual practices may have negative effects on self-esteem or lead to relationship rejections, which may then promote drug use.

Contrary to expectation, we did not find that cocaine use was associated with depression or anxiety scores, binge-eating, disordered gambling, nor self-esteem, in terms of overall group differences (Table 4). However, cocaine use was associated with significantly higher rates of ADHD, PTSD, treatment for psychological/emotional problems, and taking mental health medication(s), albeit all these results were of small effect size. It is important to consider here that many cocaine users in our sample may have had relatively low actual frequency of cocaine use, which may mask untoward metal health associations that would otherwise have been observed, such as in people with formal cocaine use disorder. The link with ADHD symptoms may fit with our earlier suggestion that impulsivity constitutes a candidate vulnerability marker for substance use problems—indeed, ADHD does show remarkably high comorbid expression with substance use disorders.^30^ ^,^^31^ The finding that cocaine use was linked with treatment for psychological/emotional problems and taking mental health medication may indicate a general relationship with psychopathology risk, even though relationships with some types of symptom were not significant in this study. The association with PTSD is in accordance with high comorbid overlap previously observed in the literature. For example, the prevalence of PTSD in cocaine users has been reported to be 8% to 43%.^32^

As can be seen in Table 5, cocaine use was associated with heightened trait impulsivity on the Barratt Impulsiveness Scale (BIS), with large effect sizes. In fact, these were the largest effect sizes observed throughout all measures of interest in this study. Trait compulsivity was not associated with cocaine use. It may be that some types of substance use are more related to compulsivity than others. For example, scores on the same compulsivity instrument have previously been shown to correlate with alcohol use, as well as other types of compulsive behaviors (such as obsessive–compulsive symptoms and disordered gambling), and with presence of substance use disorders in general.^24^ ^,^^33^ Given the established sensitivity of this instrument to compulsivity across diagnoses, our data indicate that young people who use cocaine appear impulsive but not compulsive. The potential exists that compulsivity may be more relevant to ingrained/regular habitual cocaine use, rather than occasional recreational use, in young people.

Although this is a relatively large study in a neglected area, several limitations should be considered. This was an Internet-based survey done anonymously—we feel this means participants would have felt more able to disclose their substance and mental health histories. However, online surveys have intrinsic limitations when compared to in-person clinical assessments, since the latter would yield more accurate measurements as pertains to mental health problems. Our survey response rate was 37.3%, which is relatively good and in keeping with other health surveys.^34^ ^,^^35^ However, we cannot show that these results necessarily generalize to the whole sample, an issue that applies in virtually all studies of this type. We did not differentiate between different types of cocaine use—for example, between crack cocaine use and noncrack cocaine use, which are associated with different relative risks.^3^ Our reason for not differentiating between different forms was that it is unlikely the study would have been sufficiently powered to analyze such subtypes of cocaine use; we wished instead to focus on the broader concept of cocaine use *per se.* Associations with different types of cocaine use should be examined in future work. Because we wanted to present the actual associations between cocaine use and other variables, we opted to show raw data, rather than undertake more complex analyses in this paper (such as for example having covariates). In future we hope to explore these data in a multivariate way across the full spread of substance use disorders; but we feel the current approach is valuable for the field since it shows how the different groups present in reality. This being a cross-sectional survey, causality cannot be shown. We hope that this cross-sectional study will foster interest in future longitudinal research examining impulsivity and compulsivity, and other variables, in the context of use of a variety of substances.

In summary, we found in a large sample of university students that history of cocaine use was relatively common; and had a number of associations. In particular, cocaine use was associated with more years as a student, lower grade point averages, more use of other drugs, riskier sexual practices, PTSD, ADHD, treatment for psychological/emotional problems (including taking prescribed medication), and trait impulsivity. Of these associations, the link with trait impulsivity had the largest effect sizes, highlighting the potential importance of this construct in seeking out candidate vulnerability markers for using substances such as cocaine. These results may serve to guide future longitudinal research to establish directions of effect.
